# Genetically Modified T Cells for Esophageal Cancer Therapy: A Promising Clinical Application

**DOI:** 10.3389/fonc.2021.763806

**Published:** 2021-11-09

**Authors:** Yu-Ge Zhu, Bu-Fan Xiao, Jing-Tao Zhang, Xin-Run Cui, Zhe-Ming Lu, Nan Wu

**Affiliations:** ^1^ Key Laboratory of Carcinogenesis and Translational Research (Ministry of Education), Department of Thoracic Surgery II, Peking University Cancer Hospital & Institute, Beijing, China; ^2^ Department of Thoracic Surgery, The First Affiliated Hospital of Nanchang University, Nanchang, China; ^3^ Key Laboratory of Carcinogenesis and Translational Research (Ministry of Education/Beijing), Laboratory of Biochemistry and Molecular Biology, Peking University Cancer Hospital & Institute, Beijing, China

**Keywords:** T cell receptor, chimeric antigen receptor, immunotherapy, engineered T cells, esophageal cancer

## Abstract

Esophageal cancer is an exceedingly aggressive and malignant cancer that imposes a substantial burden on patients and their families. It is usually treated with surgery, chemotherapy, radiotherapy, and molecular-targeted therapy. Immunotherapy is a novel treatment modality for esophageal cancer wherein genetically engineered adoptive cell therapy is utilized, which modifies immune cells to attack cancer cells. Using chimeric antigen receptor (CAR) or T cell receptor (TCR) modified T cells yielded demonstrably encouraging efficacy in patients. CAR-T cell therapy has shown robust clinical results for malignant hematological diseases, particularly in B cell-derived malignancies. Natural killer (NK) cells could serve as another reliable and safe CAR engineering platform, and CAR-NK cell therapy could be a more generalized approach for cancer immunotherapy because NK cells are histocompatibility-independent. TCR-T cells can detect a broad range of targeted antigens within subcellular compartments and hold great potential for use in cancer therapy. Numerous studies have been conducted to evaluate the efficacy and feasibility of CAR and TCR based adoptive cell therapies (ACT). A comprehensive understanding of genetically-modified T cell technologies can facilitate the clinical translation of these adoptive cell-based immunotherapies. Here, we systematically review the state-of-the-art knowledge on genetically-modified T-cell therapy and provide a summary of preclinical and clinical trials of CAR and TCR-transgenic ACT.

## 1 Introduction

Esophageal cancer is one of the most common cancers and a leading cause of cancer-related deaths, with its incidence and mortality increasing worldwide. Esophageal cancer can be predominantly categorized into two subtypes, esophageal adenocarcinoma, and esophageal squamous cell carcinoma (ESCC), which accounts for nearly 90% of all diagnosed patients (1). For resectable esophageal cancer, radical esophagectomy and lymph node dissection, are principal surgical treatments but also a key part of multidisciplinary therapy (2). Multi-drug chemotherapy, such as CF regimen (cisplatin and 5-fluorouracil), ECF regimen (epirubicin, cisplatin and 5-fluorouracil) combined with radiotherapy are the conventional therapies for advanced patients who cannot tolerate surgery or adjuvant treatments of resectable tumors (3). The efficacy of most molecular-targeted therapy available for esophageal cancer is suboptimal, except for the anti-HER monoclonal antibodies, trastuzumab (3). Immunotherapy is an emerging method for enhancing the anti-tumor response in patients. At present, immune checkpoint inhibitors, have demonstrated substantial clinical anti-tumor effect and tumor vaccines are under active investigation. Nivolumab alone or in combination with ipilimumab have reinvigorated the anti-tumor immune responses and increased the overall survival of esophagogastric cancer (4). Despite tremendous improvements in therapeutic modalities, the estimated overall five-year survival rate is still approximately 15% (5). Therefore, it is particularly urgent to explore effective and novel therapies to combat esophageal cancer.

**Figure 1:**
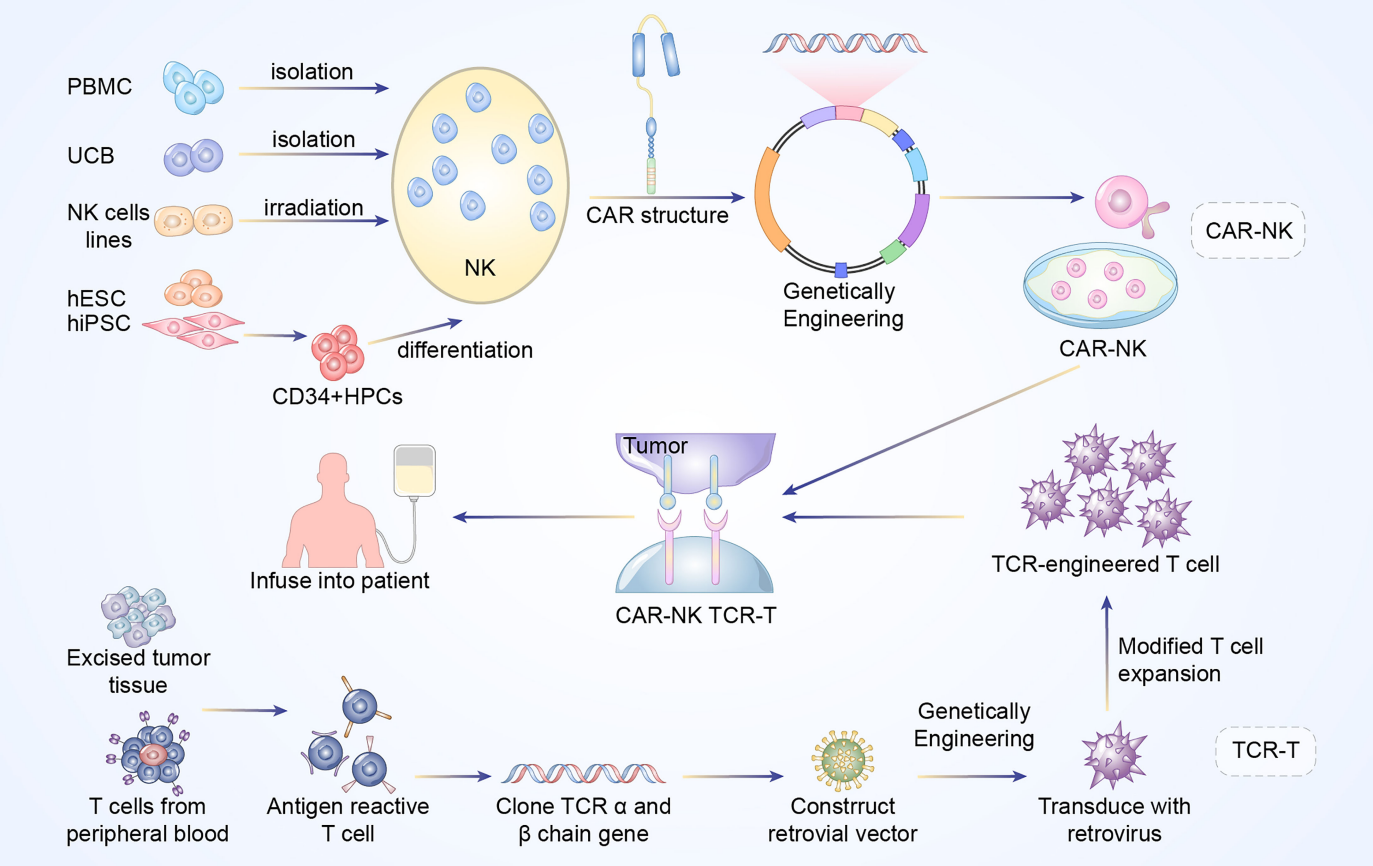
Manufacturing procedures of CAR-NK and TCR-T cells. The established NK cells principally come from PBMC, UCB, NK cell lines, ESCs, and iPSCs. The NK92 cell line after irradiation has been widely used as the main source of CAR-NK cells. In addition, primary NK cells could be extracted from peripheral blood (PB) or umbilical cord blood (UCB). The above NK cells are engineered with CAR structure to produce CAR-NK cells. Antigen reactive T cells are isolated from excised tumor tissues or PB of the patients. Subsequently, the antigen-specific TCR sequences are cloned and transfected into T cells *via* retroviral or lentivirus vector to construct TCR-engineered T cells.

**Figure 2:**
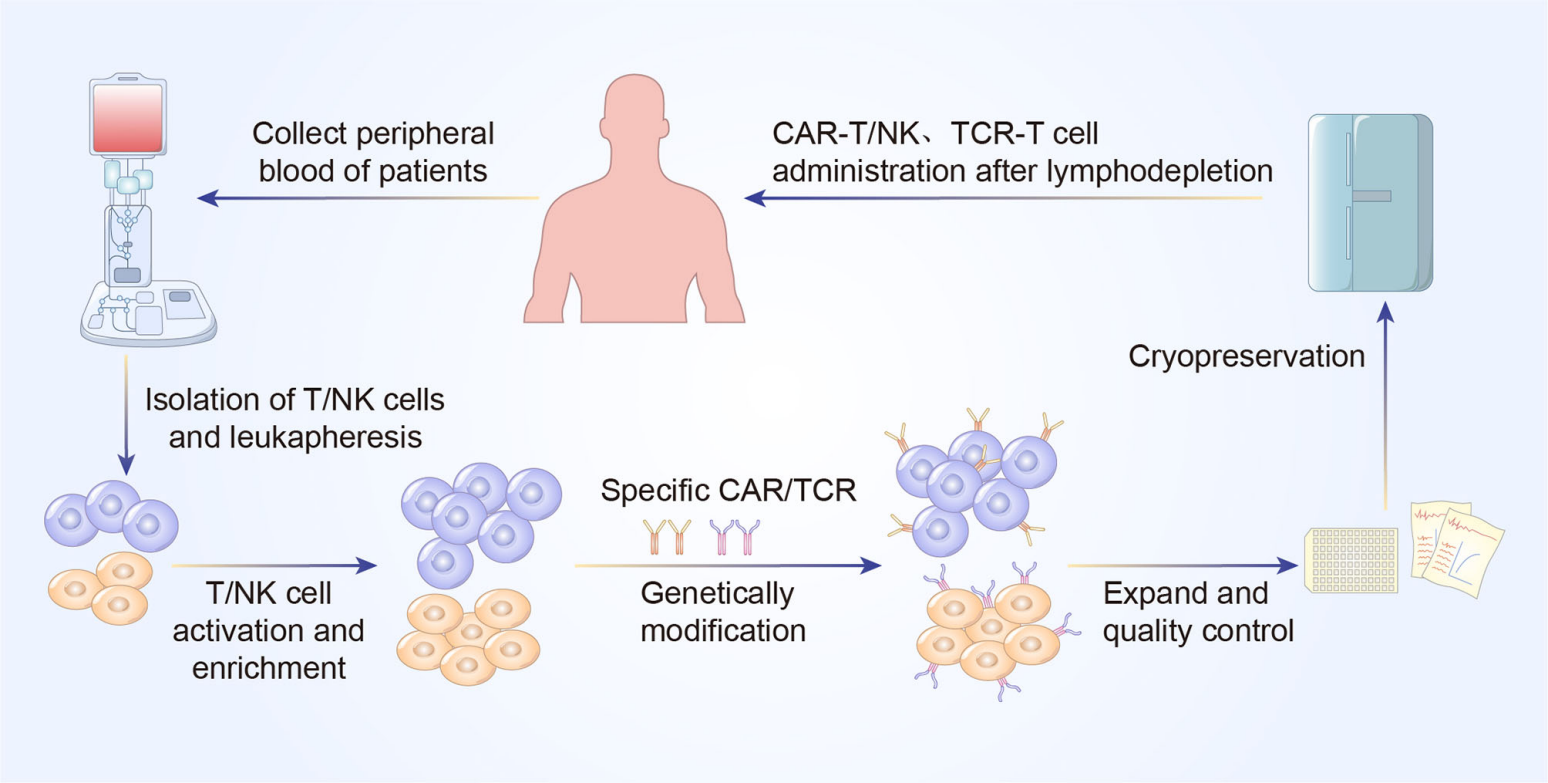
A concise workflow of genetically modified T cell therapy in clinical practice. The peripheral blood is initially collected to isolated T/NK cells. The purified T/NK cells are activated and amplified ex vivo and genetically modified to express specific CAR or TCR. Following expansion and quality control ex vivo, CAR-T/NK cells or TCR-T cells are infused back into the patients’ body to eliminate cancer cells.

CAR and TCR engineered T cell therapies, are effective and rapidly evolving immunotherapy. Typically, these adoptive T cell therapies require the patient’s own T cells to be extracted, isolated, screened, modified, expanded ex vivo, following by re-infusion back into the patients. The technique utilizes lentiviral or retroviral vector transduction to genetically modify the autologous T cells so that they could express a unique CAR or TCR with novel antigen specificity, thereby redirecting those engineered T cells to eradicate the cancer cells **(** **Figures 1** and **2** **)** (6).

## 2 CAR-T Cell Therapy: A Novel Approach for Cancer Immunotherapy

CAR-T cell therapy is an emerging curative approach against hematological tumors and has shown a satisfactory clinical response. CD19 targeted CAR-T cells have become a leading therapy against relapsed or refractory hematological malignancies, such as lymphocytic leukemia and B-cell lymphoma (7, 8). The FDA has approved four autologous CD19 targeted CAR-T cell therapy products, Tisagenlecleucel (Kymriah), Axicabtagene ciloleucel (Yescarta), Brexucabtagene autoleucel (Tecartus), Lisocabtagene maraleucel (Breyanzi) for relapsed/refractory B-cell lymphoma or acute lymphocytic leukemia, and one autologous BCMA targeted CAR-T cell therapy product, Idecabtagene vicleucel (Abecma) for multiple myeloma (7–11). The tremendous achievements of CAR-T cell therapy in treating hematological malignancies have promoted the application of this therapy to solid tumors.

### 2.1 The Design of CAR-T Structure

CARs are synthetic receptors that mainly consist of four components, extracellular domain, hinge region, transmembrane domain and intracellular signaling domain (6). Every part of the CAR structure has unique properties, and has evolved to improve the safety and optimize the cytotoxic effect of the CAR-T cells. The single chain variable fragment (scFv), is the main portion of the extracellular domain and could recognize and bind the targeted tumor specific antigens in a major histocompatibility complex (MHC)-independent manner (12). Therefore, CAR-T cells could avert the tumor immune evasion elicited by downregulation of MHC molecules. Hinge region functions to adjust the steric distance between the CAR-T cells and antigen epitopes, and the transmembrane domain can transduce extracellular antigen recognition signals into the intracellular signaling domain (13).

**Figure 3:**
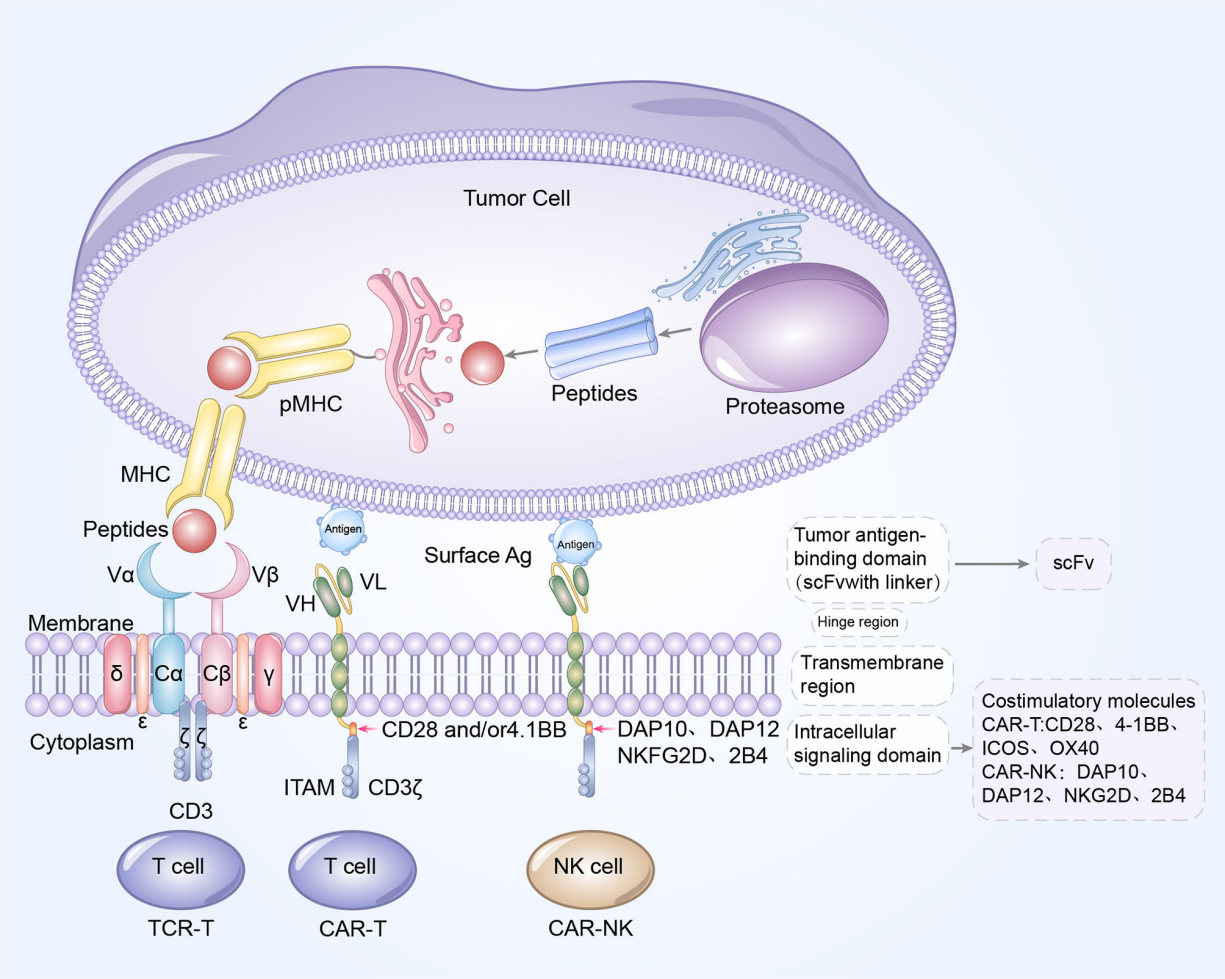
Blueprint of CAR/TCR structure. Left to right: the designs of TCR-T cell, CAR-T cells, and CAR-NK cells. CAR-T cells and CAR-NK cells share a similar CAR structure, which is composed of extracellular tumor antigen binding domain (scFv), hinge region, transmembrane region and intracellular signaling domain. The update of CAR structures is primarily reflected in the incorporation of costimulatory molecules. CD28, 4-1BB, OX40 and ICOS are the common costimulatory molecules in CAR-T cells while DAP10, DAP12, NKG2D and 2B4 are widely used costimulatory domains in CAR-NK cells. The TCR complex is a heterodimer is constituted of TCRα and TCRβ chains, noncovalently connected to the three CD3 dimers (CD3γϵ, CD3δϵ, and CD3ζζ). Antigen peptides from tumor cells bind to MHC molecules to form pMHC, and subsequently TCR recognizes and binds to the antigens presented by pMHC. The extracellular antigen signaling is transduced by TCR-CD3 complex into intracellular signaling, and mediates the elimination and killing of tumor cells.

The intracellular signaling domains of different generations are distinct from each other. First- generation CARs only contain a single signaling molecule CD3ζ, while second- and third- generation CARs have incorporated one and two costimulatory molecules respectively (**Figure 3**). The costimulatory domains of CAR-T cells, primarily include 4-1BB, CD28, OX40, ICOS, CD27, MYD88, CD40, DAP12A, among which, 4-1BB and CD28 are most widely studied and have been approved for use by FDA (14). CAR-T cells with CD28 costimulatory molecule, demonstrated a rapid antitumor activity but a decreased persistence, compared to 4-1BB (15). The remaining costimulatory molecules have only been validated to be efficacious in preclinical evaluation, whereas have not been clinically evaluated. More recently, the next generation of CAR-T cells is ongoing active investigation in order to better support the anti-tumor effect of CAR-T cells. Armored CAR-T cells are being modified to generate cytokines, chemokines, or co-expressing immunomodulatory ligands to overcome the immunosuppressive tumor microenvironment (TME) and sustain the function of CAR-T cells (14). CAR-T cells that secrete immunomodulatory cytokines, which is also known as T cells that redirect general cytokine-mediated killing, are an example of armored CAR-T cells. CAR-T cells with inducible proinflammatory cytokines IL-12 or IL18 secreting, could alter the immunosuppressive milieu by redirecting more immunes cells into tumor sites, which have showed an enhanced cytotoxicity in solid tumors (16). Additionally, IL-7 and CCL19 expressing CAR-T cells have exhibited an augmented infiltration and proliferative competence *in vitro* and *in vivo*, compared with convention CAR-T cells (17). Gene editing technology could mediate the knockdown of TCR α/β chains, to generate the next generation universal CAR-T cells with higher safety, thereby averting graft-versus-host disease (GVHD) (18, 19). Tandem CAR-T cells, equipped with two scFvs, simultaneously target two tumor antigens and therefore, could overcome the anti-tumor immune escape. For example, CD70 and B7-H3 targeted tandem CAR-T cells have demonstrated efficacious against esophageal cancers in preclinical models (20).

### 2.2 Esophageal Cancer−Associated Antigens for CAR−T Cell Therapy in Preclinical Studies

One hurdle in applying CAR−T cell therapy against solid cancers is the paucity of targeted antigens. Since a mature manufacturing process of CAR-T cell therapy is already available, the identification of specific antigens for optimal targeting is imperative for expanding its application.

The ideal antigens for CAR-T cell therapy should have high specificity and high coverage of tumor cells to ensure both safety and efficacy (21). Indeed, several surface antigens have already been identified in preclinical studies as potential targets for CAR-T cell therapy against esophageal cancer as described below.

#### 2.2.1 Human Epidermal Growth Factor Receptor 2

HER2 belongs to HER/ErbB family of receptor tyrosine kinases, which represents a crucial therapeutic strategy in HER2-postive Esophageal cancer (22). In recent studies, HER2-based CAR-T cells have been established and their effectiveness in esophageal cancer treatment has been investigated both *in vitro* and *in vivo*. These cells can recognize and eliminate the HER2-amplied ESCC cell lines, ECA109 and TE-1, elevate the levels of proinflammatory cytokines and moreover, intratumor administration of those modified T cells significantly inhibited the tumor growth in ECA109 xenografts (23).

#### 2.2.2 Erythropoietin-Producing Hepatocellular Receptor A2

EphA2 is a member of the Eph family, which serves as a surface antigen for its carcinogenic effects. EphA2 is highly expressed in tumor cells, while at relatively low levels in most normal adult tissues, which suggested its potential use in cancer immunotherapy (24). The second generation of EphA2 targeted CAR-T cells exhibited an obviously inhibitory effect on ESCC cells in a dose-dependent manner (25). Moreover, similar researches have been conducted on other solid tumors, such as central nervous system tumors (26). Thus, further investigation to evaluate the efficacy of EphA2 targeted CAR-T cell therapy against esophageal cancer is warranted.

#### 2.2.3 Mucin-1

MUC1 is a glycoprotein whose expression levels are aberrantly upregulated in various carcinomas, such as esophageal cancer (27). MUC1 targeted CAR-T cells were verified to exert substantial cytotoxicity effects on solid malignancies, including triple-negative breast cancer and pancreatic cancer (28, 29). However, the efficacy of CAR-T cell therapy against solid tumor was less satisfactory, which could be partially attributed to the immune suppression of TME and loss of CAR-T cell antitumor function. To address this issue, enhanced CAR-T cells that contain a JAK-STAT signaling domain has been developed. The engineered MUC1 targeted CAR-T cells activated the JAK-STAT pathway, secreted higher level of cytokines, showed superior proliferative capacity and persistence, and mediated greater antitumor activity both in subcutaneous xenograft tumors and a PDX mouse model of esophageal cancer (30). In summary, directly integrating the cytokine receptor domain into CARs could be a potential strategy to improve the efficacy of CAR-T cell therapy.

#### 2.2.4 CD276 (B7-H3)

CD276 (also named B7-H3), belongs to B7 superfamily molecules. It is an immune checkpoint and could elicit similar inhibitory activity as PD-1 on T cells (31). It is overexpressed in a wide range of cancers and associated with poor prognosis of human patients, which makes it an appealing target for CAR-T cell therapy (31). CD276 specific CAR-T cells efficiently eliminated ESCC cells both *in vitro* and *in vivo* (32). Tandem CAR-T cells targeting CD276 and CD70 also exerted enhanced tumoricidal activity against multiple solid tumors (20). These findings indicated that CD276-targeting CAR-T cells merit further testing in ESCC clinical trials.

### 2.3 Clinical Trials

CAR-T cell therapy has revolutionized the treatment of B cell-derived hematological malignancies, although the responses and results are less favorable in solid cancers. The high heterogeneity of solid tumor cells, paucity of targeted antigens, on-target, off-tumor toxicity, immunosuppressive TME, inefficient trafficking and transient persistence of CAR-T cells make it more complex to treat the solid tumors with CAR-T cell therapy (33). Currently, substantial effort is being invested in enhancing the efficacy of CAR-T cell therapy against solid cancers, hopefully to rejuvenate the landscape of immunotherapy. Some targeted antigens are being investigated and evaluated in clinical trials of CAR-T cell therapy against esophageal cancer (**Table 1**).

**Table 1:** Summary of ongoing clinical trials of gene-modified T-cell therapy in the treatment of esophageal cancer.

Immunotherapeutic strategy	Targeting antigen	Clinical Trial	Sponsor	Estimated Enrollment	Phases	Status
CAR-T cell therapy	MUC1	NCT03706326	The First Affiliated Hospital of Guangdong Pharmaceutical University	20	Phase I	Recruiting
CAR-T cell therapy	HER2	NCT03740256	Baylor College of Medicine	45	Phase I	Recruiting
CAR-T cell therapy	EpCAM	NCT03013712	First Affiliated Hospital of Chengdu Medical College	60	Phase I/II	Unknown
CAR-T cell therapy	Claudin18.2	NCT04581473	Carsgen Therapeutics, Ltd.	102	Phase I/II	Recruiting
CAR-T/TCR-T cell therapy	NY-ESO-1	NCT03941626	Shenzhen BinDeBio Ltd.	50	Phase I/II	Recruiting
CAR-T/TCR-T cell therapy	NY-ESO-1	NCT03638206	Shenzhen BinDeBio Ltd.	73	Phase I/II	Recruiting
TCR-T cell therapy	NY-ESO-1	NCT03159585	Zhujiang Hospital	6	Phase I	Completed
TCR-T cell therapy	NY-ESO-1	NCT02869217	University Health Network, Toronto	22	Phase I	Recruiting
TCR-T cell therapy	NY-ESO-1	NCT02366546	Mie University	9	Phase I	Unknown
TCR-T cell therapy	MAGE-A3	NCT01273181	National Cancer Institute (NCI)	1	Phase I/II	Terminated
TCR-T cell therapy	MAGE-A4	NCT03132922	Adaptimmune	52	Phase I	Recruiting
TCR-T cell therapy	MAGE-A4	NCT02096614	Mie University	18	Phase I	Completed
CAR-NK cell therapy	PD-L1	NCT04847466	National Cancer Institute (NCI)	55	Phase II	Not yet Recruiting

## 3 CAR-NK Cell Therapy: A Professional Killer in Next-Generation Immunotherapy

With the unprecedented advances in CAR-T cell therapy, there is also an increasing interest in constructing CAR-natural killer (CAR-NK) cells for cancer therapy. Natural cytotoxicity receptors on NK cells (NKp30, NKp44 and NKp46), and other receptors such as NKG2D and DNAM-1, can recognize and interact with specific ligands on tumor cells, so that NK cells could directly exert a cytotoxic effect without MHC presentation (34). CAR engineering enhances the specificity of NK cells by equipping them with more effective weapons to fight cancer cells. The combination of intrinsic and engineered killing potency gives CAR-NK cell therapy great promise for enhancing cancer immunotherapy.

Although CAR-T cell therapy has achieved impressive antitumor efficacy, it still has several limitations, including on-target/off-tumor toxicity, severe cytokine storm and neurotoxicity (35). CAR-NK cells have aroused considerable interest in cancer treatment, primarily attributable to their potential to circumvent these obstacles, while exhibiting a potent antitumor effect (36). Specifically, due to the transient lifespan of CAR-NK cells, they have relatively low impact on normal tissues, and CAR-NK cells will not induce on-target/extra-tumor damage like CAR-T cells. Furthermore, the administration of CAR-NK cells is less likely to cause serious cytokine release syndrome or neurotoxicity, as significantly elevated serum proinflammatory cytokines (such as IL-6) were not observed in patients (37). Although this is conducive for improving safety, it does restrain the efficacy of CAR-NK cell therapy, since they are susceptible to exhaustion due to lack of cytokine support. In addition, the challenges, which impede the application of CAR-T cell therapy, also hinder the development of CAR-NK cell therapy, such as low infiltration into tumor sites and the immunosuppressive effect of TME.

### 3.1 The Design of CAR-NK Cell Structure

A comprehensive understanding of the immunological function of NK cells and potential mechanisms has prospered the development of CAR-NK therapy. The CAR-NK cells share a similar CAR structure with CAR-T cells, which consist of an extracellular antigen recognition and binding domain (scFv), an extracellular hinge region, a transmembrane domain and an intracellular signaling domain. The targeted antigen recognition of CAR-NK cells is MHC-independent, making it possible to establish NK cell banks, rather than constructing individualized CAR-NK cells (38).

As previously discussed, the updates in CAR design are principally reflected in the inclusion of costimulatory molecules. Among them, the immunoglobulin superfamily member CD28, and TNF receptor superfamily member 4–1BB have been the most explored. However, the role of costimulatory molecules, CD28 in CAR-NK cell signaling remains unclear since it is not typically expressed in NK cells, which encourages the researchers to elucidate other costimulatory domains with greater therapeutic specificity for NK cell signaling, such as DAP10, DAP12 or 2B4 (**Figure 3**) (39). 2B4 is a specific costimulatory receptor activating the NK cells and a member of signaling lymphocytic activation molecule family (40). CAR-NK cells with an NKG2D transmembrane domain and 2B4 costimulatory domain have exhibited extraordinary anti-tumor activity in solid tumors as well as hematologic malignancies (40, 41). As further research is conducted in the future, CAR-NK cell therapy may markedly change the landscape of cancer immunotherapy.

### 3.2 Current Preclinical and Clinical Trials of CAR-NK Therapy

Abundant preclinical and clinical evidence has confirmed the feasibility of utilizing CAR-NK cells to combat lymphocytic leukemia, lymphoma, and multiple myeloma (42, 43). For solid malignancies, HER2 targeted CAR-NK-92 cells have been reported to inhibit the growth of breast and ovarian cancer cells (44). Moreover, ongoing clinical trials using targeted CAK-NK cells on solid tumors are assessing the antitumor activity of MUC1 (NCT02839954), mesothelin (NCT03692637), NKG2D (NCT03415100), prostate specific membrane antigen (NCT03692663), ROBO1(NCT03940820) and HER2 (NCT03383978). However, there are almost no reported preclinical or clinical trials of CAR-NK therapy against esophageal cancer, probably due to the lack of specific tumor-targeted antigens for esophageal cancer. Moreover, the existing animal models fail to simulate the TME that would accurately assess the function of CAR-NK cells (39).

## 4 TCR-T: A Promising Alternative to Traditional Immunotherapies

Considerable advances in genomics and next-generation sequencing technologies have made it possible to identify the sequences of tumor specific TCRs and establish specific genetically modified T cells to fight cancers (45). One successful example is the application of TCR-T therapy. The emerging TCR-T cell therapy utilizes isolated TCR-encoding genes from tumor reactive T cells, and subsequently, transduces these TCR sequences into unmodified T cells to manufacture specific TCR-T cells to eradicate targeted cancer cells. Essentially, the engineered TCR-T cells are tumor antigen-specific T cells, and therefore, these cells could recognize and bind to the targeted tumor antigens, in a manner similar to unmodified T cells (**Figure 3**).

TCR-T therapy is another innovative and effective genetically modified T cell immunotherapy in addition to CAR-T cell therapy and CAR-NK therapy. Even though CAR-T cell therapy has achieved encouraging progress in treating hematologic malignancies, it still fails to restrain the progression of most solid tumors. In contrast, TCR-T cell therapy has demonstrated remarkable potential in treating solid tumors (46).

### 4.1 The Design of TCR-T Structure

TCR is the primarily antigen-recognition domain. It is a heterodimer consisting of an α chain and a β chain, each of which contain a variable region and a constant region. TCRs must be matched with human leukocyte antigen (HLA) alleles before they recognize and bind to specific antigens presented by peptide-MHC (pMHC) to effectively eliminate or reduce tumor cells, which is significantly distinct from the mechanism of CAR-T cells (**Table 2**) (47). Antigens within any subcellular compartment, once processed and presented by MHC molecules, can be recognized by TCR-T cells. Therefore, TCR-T cell therapy has a wider range of targeted antigens, whether they are extracellular antigens or intracellular antigens. Furthermore, genetic modification has increased the affinity for cancer cells and TCRs, enabling TCR-T cells to better recognize intracellular antigens (33). TCR antigen recognition signaling is transduced *via* the TCR-CD3 complex, which is primarily composed of α chain and β chain of the TCR, noncovalently connected to the CD3 dimers CD3γϵ, CD3δϵ, and CD3ζζ (**Figure 3**) (48).

**Table 2:** Comparison of structural features and mechanisms of TCRs versus CARs in cancer immunotherapy.

Property	TCR	CAR
Receptor structure	α and β chain, CD3	ScFv, CD3ζ, costimulatory molecules
Subunits	10	1
Antigen recognition domain	TCR	ScFv
Antigen recognition	Extracellular and intracellular antigen	Extracellular antigen
MHC dependence	MHC-dependent	MHC-independent
Receptor affinity	Micromolar range	Nanomolar range
Antigen epitope density	One	Several orders of magnitude
Dosage of transfusion cells	high	low

### 4.2 Current Preclinical and Clinical Trials of TCR-T Therapy

TCR-T cell therapy is a promising modality for cancer treatment and initiates an era of highly personalized and precise cancer therapy. In recent decades, numerous studies have investigated the effectiveness and safety of the emerging therapeutic strategy. It is worth noting that recently our research group successfully attempted to identify four latent membrane protein-2A (LMP2A)-specific TCRs and confirmed the cytolytic activity of LMP2A targeted TCR-T cells against Epstein–Barr virus latency II tumors both *in vivo* and *in vitro*. Our updated findings revealed that LMP2A -specific TCR-T cells could be a novel alternative for patients with EBV-associated malignancies regardless of specific HLA type and epitope (49).

Unlike CAR-based ACTs, a large percentage of TCR-T cell therapy clinical trials mainly focused on solid tumors instead of hematological malignancies, though TCR-T cell therapy might seem promising for liquid tumors (50). Clinical trials of TCR-T cell therapy have exhibited satisfactory results in solid tumors, including esophageal cancer. Below, we focused on TCR-T cells, which mainly target cancer testis antigens, including NY-ESO-1, MAGE-A3, MAGE-A4, and systematically reviewed the relevant preclinical and clinical studies of TCR-T therapy against esophageal cancer.

#### 4.2.1 New York Esophageal Squamous Cell Carcinoma 1

NY-ESO-1 is a well-known cancer testis antigen expressed during the early stages of fetal development. Its expression declines drastically after birth and is undetectable in healthy adult tissues (51). Therefore, it could be a potential candidate for ACT against NY-ESO-1 positive tumors. In fact, it is the most targeted antigen in TCR-T based clinical trials. Interestingly, preclinical evidence has demonstrated that NY-ESO-1 targeted TCR-T therapy has a long-term antigen-specific tumoricidal effect on glioblastoma cells *in vitro* (52). Tumor regression and extended overall survival were also observed in neuroblastoma-bearing xenograft mouse studies after treatment with NY-ESO-1 targeted TCR-T cells (53).

The safety and feasibility of NY-ESO-1 targeted TCR-T therapy were further investigated in phase I/II clinical trials. Clinical trials were performed in a broad range of solid tumors, as well as in some liquid tumors. One trial reported that 37 out of 42 patients with synovial sarcoma (NCT01343043) benefited from NY-ESO-1 targeted TCR-T therapy and concluded that lymphodepletion may improve the engineered TCR-T cell persistence and therapeutic efficacy (54). NY-ESO-1 targeted TCR-T therapy also achieved encouraging clinical responses in patients with advanced multiple myeloma, with no lethal adverse reactions occurring (55). Detailed information about NY-ESO-1 targeted TCR-T therapy against esophageal cancer (NCT03941626, NCT03638206, NCT03159585, NCT02869217, NCT02366546) is summarized in **Table 1**. Owing to its substantial efficacy and controllable side effects, NY-ESO-1 has been considered as one of the optimal TCR-T therapy targets for solid tumors.

Enhanced persistence and function of TCR-T cells are related to the effectiveness of TCR-T therapy. Corresponding strategies to improve proliferative activity include multiple repetitions of TCR-T cell infusions and combinatorial application with immune checkpoint inhibitors (56). Other strategies include the transduction of artificial T cell-activating adapter molecules (ATAMs) to extend the persistence of TCR-T cells. ATAMs are generated by inserting the intracellular domain of CD28 or 4-1BB into CD3ζ, which regulates downstream intracellular signaling following antigen stimulation (57). The superior proliferative capability and antitumor effect of NY-ESO-1 targeted TCR-T cells transduced with ATAM were confirmed both *in vitro* and in a mouse xenograft model (57, 58).

#### 4.2.2 Melanoma-Associated Antigen-A Family

MAGE-A3 and MAGE-A4 are two members of MAGE-A subfamily, and their overexpression is associated with poor prognosis (59). They are also cancer-testis antigens, whose expression is restricted to immune-privileged sites in normal tissues (60). Moreover, the expression of MAGE-A3 and MAGE-A4 is upregulated in multiple malignancies, including esophageal cancer, which provides a theoretical basis for their application in TCR-T therapy (61). Studies have validated the antitumor activity of MAGE-A4 specific TCR cells since they could suppress the growth of MAGE-A4 expressing tumors, such as esophageal cancer and lung carcinoma (62).

In a clinical trial, a patient who received MAGE-A3 specific TCR-T cell therapy had a preliminary remission of esophageal cancer at 1 month, but suffered a serious tumor progression at 2 months and died shortly thereafter (63). Similarly, partial responses of MAGE-A3 specific TCR-T cell therapy against esophageal cancer were observed in another clinical trial, but disease progression still occurred at 4th month (64). The first-in-man clinical trial of MAGE-A4 specific TCR-T cell therapy in patients with recurrent esophageal cancer demonstrated the safety and long persistence of TCR-T cells (65). Thus, the efficacy and feasibility of MAGE-A specific TCR-T therapy is still questionable based on the existing evidence, and more related studies are required to further validate the possibility of applying MAGE-A specific TCR-T therapy to treat esophageal cancer.

## 5 Concluding Remarks and Outlook

Genetically engineered T cell therapies, including CAR-T cell therapy and TCR-T cell therapy, have revolutionized the immunotherapy of several hematologic malignancies. As intense researches continue to deepen in recent decades, these technologies represent a breakthrough in adoptive T cell therapy for advanced solid malignancies. In the meantime, NK cells have become an effective and reliable CAR engineering platform. One of the commonalities of these three novel treatment options is to empower the patient’s own immune system to recognize specific antigens and subsequently eradicate the tumor cells.

In this mini-review, we discuss the structure and design of CARs and TCRs, which are structurally and functionally distinct receptors. The scFv, derived from antibodies, is the main extracellular domain of CARs. It recognizes and binds to the targeted antigens, which are neither restricted and nor dependent on MHC molecules. TCR, composed of an α chain and a β chain, is the main antigen-recognition domain of TCR-T cells, and capable of binding to specific antigens presented by pMHC. CARs could only target cell surface antigens, so that the intracellular tumor antigens are mostly inaccessible to CAR-based therapies. In contrast, TCR-T therapy has evolved to recognize both intracellular antigens and cell surface antigens (66). In addition, we summarize the up-to-date preclinical and clinical trials of esophageal cancer associated antigen targets in CAR-T cell therapy and TCR-T cell therapy, although studies on esophageal cancer remain in their infancy. Currently, the potential targets against esophageal cancer include MUC1, HER2, EpCAM, EpA2 and CD276, for CAR-T cell therapy, NY-ESO-1, MAGE-A3 and MAGE-A4 for TCR-T cell therapy. Identification and development of novel targeted antigens for genetically engineered T cell therapies are imperative for treating patients with solid malignancies.

T cells can be divided into two types, based on the composition of TCR: αβT cells and γδT cells. Current studies of TCR-T cells are predominantly focused on αβT, and the conventional αβ-TCR-T cells have been described above. γδT cells with TCR comprised of γ and δ heterodimer chains, are another subpopulation of T cells, only represents 1-5% of peripheral blood T cells (67). γδT cells, endowed with both innate and adaptive immunity, have exhibited potent tumor recognition and elimination effect on various tumors. Unlike αβT cells, γδT cells do not usually express CD4 or CD8 molecule, and they could recognize the antigens in an MHC independent manner, and therefore, are insensitive to immune escape mediated by loss of targeted antigens (68). Engineered γδT cells have exhibited an equivalent cytotoxicity, while a decline in the release of cytokines, compared to conventional αβT cells with identical TCR transferred (69). Furthermore, the γ and δ chains of γδT cells would not mismatch with transferred α or β chains of TCR, thereby preventing the arise of self-reactive TCR clones and increasing the safety of genetically modified T cell therapy. Therefore, γδT could overcome the limitations of conventional TCR-αβT and serve as an alternative candidate in genetically modified T cell therapy. Although the preclinical studies have revealed the evaluated the prognostic role of γδT cell therapy in cancer therapy, the divergences in genes encoding γ and δ chains of TCR between rodents and primates make it difficult to provide evidence for applying γδT cell immunotherapy in cancer patients (70). Besides, the evaluation of the adverse effect of γδT cell therapy was failed to be implemented either for the mouse cells lacked the potentially relevant human self-antigens (71). In conclusion, future efforts such as 3D organoid culture to mimic the *in vivo* microenvironment will provide more convincing proof for γδT cell therapy.

Although these immunotherapies have achieved impressive results in combating liquid tumors, they still face multiple obstacles and challenges in treating solid cancers, such as on-target/off-tumor toxicity, severe treatment-related toxicities, cytokine storm, neurotoxicity, GVHD, hostile TME, identification of ideal antigens, tumor immune evasion, limited tumor infiltration levels and exhaustion of T cells (14, 33). The researchers should give priority to the management of diverse toxicities, which have aroused widespread concern. Once administered into the patients’ blood, the activity of CAR-T cells is uncontrollable. Therefore, suicide switches, have been incorporated into CAR-T cells to decrease the toxicities of the treatment, which could be activated to selectively eradicate the CAR-T cells in case that severe adverse effects occur. HSV-TK, a well-characterized suicide gene, could phosphorylate the ganciclovir (GCV) into GCV-triphosphate, which subsequently inserted into DNA to disrupt DNA synthesis and eventually induce the cell death of CAR-T cells. The feasibility and efficacy of CD44v6 targeted CAR-T cells with HSV-TK suicide gene have been verified in preclinical studies of acute myeloid leukemia and solid tumors (72, 73). Other suicide switches include inducible caspase-9, which could be dimerized to activate the downstream intracellular apoptotic signaling, thereby eliminating the CAR-T cells (74). The inhibitory chimeric antigen receptors contain a PD-1 or CTLA-4 based inhibitory domain, which could shield the normal cells from being killed by specific CAR-T cells, to overcome the on target, off tumor toxicities (75). We believe that the immunosuppression of TME could be overcome with gene editing tools and combined therapies that could protect the CAR-T (CAR-NK or TCR-T) cells from inhibition of TME (76). As for antigen identification, the patient-specific neoantigen targets are of high safety, and have attracted great interest (77). To prolong the persistence of CAR-T cells, dual-receptor CAR-T cells have been designed to express two synthetic receptors simultaneously, one for recognizing targeted antigens, and the other promoting the proliferation of T cells (33). Further efforts are still urgently required to achieve the full potential of these three emerging ACT.

## Author Contributions

NW contributed significantly to fund support and the conception of the review. YG-Z and BF-X, and JT-Z contributed to wrote the manuscript. XR-C contributed to prepare and revise the manuscript. ZM-L helped proposed some constructive suggestions. All authors contributed to the article and approved the submitted version.

## Funding

This work was supported by the National Key Research and Development Program of China (No. 2018YFC0910700), Beijing Human Resources and Social Security Bureau (Beijing Millions of Talents Project, 2018A05), Beijing Municipal Administration of Hospitals’ Youth Programme (QMS20191107), National Natural Science Foundation of China (No. 81972842), Beijing Natural Science Foundation (No. 7192036), Natural Science Foundation of Jiangxi Province (20202BABL206088).

## Conflict of Interest

The authors declare that the research was conducted in the absence of any commercial or financial relationships that could be construed as a potential conflict of interest.

## Publisher’s Note

All claims expressed in this article are solely those of the authors and do not necessarily represent those of their affiliated organizations, or those of the publisher, the editors and the reviewers. Any product that may be evaluated in this article, or claim that may be made by its manufacturer, is not guaranteed or endorsed by the publisher.
